# Sleep, Emotion, and Learning Across the Lifespan: Insights into Cultural Factors and Biological Mechanisms

**DOI:** 10.3390/brainsci16090937

**Published:** 2026-09-01

**Authors:** Anne M. Fink

**Affiliations:** Department of Biobehavioral Nursing Science, College of Nursing, University of Illinois Chicago, Chicago, IL 60612, USA; afink2@uic.edu

Sleep is essential for happiness, creativity, and success—but people often find it difficult to prioritize sleep. Insufficient sleep can be a serious problem for school-age children and young adults who commonly stay up late to study and use technology. Consequently, the term “screen-time” has evolved to describe the prevalent nocturnal use of digital devices, which can have serious impacts on the nervous system and ability to achieve restful sleep. In the Special Issue, “Relationships Between Disordered Sleep and Mental Health,” scientists around the world contributed findings about the complex interactions that link sleep, stress, emotion, and cognition (contributions 1–6). Authors explored mechanistic hypotheses by analyzing heart rate variability (HRV) and microbiome and metabolome biomarkers (contributions 1,2). Importantly, this collection of research reports and a systematic review identified important global themes about sleep and mental health across the lifespan, focusing on challenges faced by children, adolescents, and young to mid-life adults (contributions 1–6). The compilation sparks interesting debates about potential therapeutic targets that could restore healthier sleep, thereby optimizing scholastic performance and mental health. The editorial highlights key observations from the Special Issue.

## 1. Students Are Vulnerable to Poor Sleep and Digital Device Addiction

A prevailing theme in the Special Issue focused on students’ struggles to obtain adequate sleep. For example, Wang et al. and Whitehead and Horton found that university students had median scores on the Pittsburgh Sleep Quality Index that fell into the moderate to poor sleep quality range (contributions 3,4). Mamun et al. reported that high school students experienced significantly greater daytime sleepiness when they had televisions in their bedrooms and used smartphones or gaming gadgets at night (contribution 5). In a systematic review, Fink and Kerulis demonstrated how elite athletes learned to prioritize sleep (routinely sleeping 6 to 8 h per night); however, their sleep quality and athletic performances were still adversely affected by stress, early morning practice sessions, late night games, and jet lag (contribution 6). Across the studies, symptoms of depression and anxiety were significantly associated with sleep-related problems, such as longer sleep onset latency (contributions 3,4), shorter sleep durations (contribution 3), and daytime dysfunction (contributions 3,5).

## 2. Cultural Awareness and Demographic Characteristics Are Important

All six articles demonstrated the importance of understanding cultural and demographic factors, such as socioeconomic resources, lifestyles, and the potential roles of sex or gender (contributions 1–6). In a study of health science university students in China, for example, Wang et al. proposed culturally relevant definitions of positive versus negative affect to ensure construct validity (contribution 3), demonstrating how emotional variables are context-specific. In Bangladesh, Mamun et al. found that excessive daytime sleepiness in adolescents was significantly predicted by living in urban settings, higher family incomes, and female sex (contribution 5), emphasizing the importance of studying the environment to understand sleep-related behaviors. Furthermore, scientists require knowledge about research participants’ cultural and religious values to accurately examine sleep and mental health. The systematic review about student athletes demonstrated how religious observances (e.g., Ramadan) could alter athletes’ sleep schedules; however, this did not impair athletic performance when coaches adjusted training and competition schedules to give athletes sufficient time for sleep (contribution 6).

## 3. Biological Metrics Could Elucidate New Avenues for Therapeutic Interventions

Two studies focused on determining biological mechanisms linking sleep and cognition and mental health (contributions 1,2). Komóczi et al. measured HRV in university students while they performed a series of gamified cognitive experiments (e.g., memory and driving tests). Subjects who reported ‘feeling rested’ achieved higher cognitive performance scores on the tests. In terms of HRV parameters, cognitive performance was negatively associated with the root mean square of successive differences (RMSSD, a marker reflecting parasympathetic nervous system activity) and positively associated with the Baevsky Stress Index, suggesting that mild arousal enhanced performance (contribution 1). These observations require further investigation to determine whether HRV could be used to assess and optimize student health and performance. In the collection of articles, Fink and Kerulis also described how athletic coaches may use HRV metrics to guide student athletes’ training and rest schedules in the future (contribution 6).

Using 16S rRNA gene sequencing, Ahmad et al. studied the gut microbiome during a 12-week trial to determine the effects of probiotic supplements containing Lactobacillus and Bifidobacterium strains (contribution 2). Compared with placebo, the supplement was associated with improved sleep efficiency, shorter rapid eye movement (REM) sleep latency, decreased anxiety and depression scale scores (Generalized Anxiety Disorder-7 and Patient Health Questionnaire-9), and increased fecal sample microbial diversity (Shannon index). Ahmad et al. concluded that using therapies to manipulate the gut microbiome may be a promising intervention to improve sleep and mental health; they hypothesized roles for the kynurenine pathway and neurotransmission during REM and non-REM sleep to explore in future studies (contribution 2).

In conclusion, the Special Issue “Relationships Between Disordered Sleep and Mental Health” demonstrated multifaceted relationships between sleep and mental health, especially in children and young adults. Digital distractions may profoundly impair sleep, delay bedtimes, and disrupt circadian rhythms [1], emphasizing the importance of pediatric guidelines that recommend healthy limits on technology utilization in school-age children. The literature reviewed in the Special Issue also illustrated insights for enhancing the rigor of future research. For example, controlling for confounding variables (e.g., dietary intake) and understanding cultural norms is critical for designing reliable metrics and valid studies. Importantly, two potential avenues for future therapies emerged in the Special Issue. Considering that the gut microbiome synthesizes neurotransmitters that support mood, sleep, and cognition (e.g., serotonin, gamma-aminobutyric acid, and dopamine), advancing knowledge about microbial and metabolic pathways is crucial. Dietary, probiotic, and prebiotic interventions may restore microbial balance, thereby improving sleep and mental health, but the cause-and-effect mechanisms should be rigorously investigated. Because circulating and excreted concentrations of metabolites are affected by numerous covariates (e.g., diet, sleep, hormones, medications), it is important to consider these aspects in future research designs [2].
